# Metabolic and Histopathological Effects of Fructose Intake During Pregestation, Gestation and Lactation in Rats and their Offspring

**DOI:** 10.4274/jcrpe.1776

**Published:** 2015-03-05

**Authors:** Erkan Sarı, Ediz Yeşilkaya, Ahmet Bolat, Turgut Topal, Bilal Altan, Kürşat Fidancı, Mehmet Saldır, Galip Erdem, Mustafa Gülgün, Yasemin Gülcan Kurt, Ahmet Güven

**Affiliations:** 1 Gülhane Military Medical Academy, Department of Pediatric Endocrinology, Ankara, Turkey; 2 Gülhane Military Medical Academy, Department of Physiology, Ankara, Turkey; 3 Gülhane Military Medical Academy, Department of Pediatric Surgery, Ankara, Turkey; 4 Gulhane Military Medical Academy, Department of Biochemistry, Ankara, Turkey

**Keywords:** Fructose, adipose, liver, kidney, metabolic effect, histopathological effect, rat offspring

## Abstract

**Objective:**

Studies have demonstrated a significant relationship between maternal fructose intake and metabolic outcome in their offspring. However, there is a paucity of data about the long-term effects of fructose intake on the offspring of fructose-fed dams. Therefore, we planned a study to evaluate the long-term effects of fructose intake on the offspring of dam rats fed a high-fructose diet.

**Methods:**

Sixteen virgin female Sprague-Dawley rats were divided into two groups. Group 1 received a regular diet and Group 2 a high-fructose diet. Both groups received their experimental diets for 8 weeks before conception. They were mated and continued to feed with their experimental diet during mating and during their pregnancy and lactation periods. After weaning, the offspring from each group were divided into two groups. Group 1A received a regular diet, Group 1B - a fructose diet, Group 2A - a regular diet and Group 2B received a fructose diet. After weaning, the offspring were anesthetized and blood samples were collected for biochemical analysis. Liver, kidney and retroperitoneal adipose tissue were harvested for histopathological examination. Primary antibodies against inducible nitric oxide synthase (iNOS) and cyclooxygenase-2 (COX-2) were determined as early inflammation markers.

**Results:**

After weaning, while daily water consumption was found to be significantly higher in Groups 2B and 1B (p<0.01), daily laboratory chow consumption was significantly lower in Groups 1A and 2A (p<0.01). Body weight was significantly higher in Groups 1B and 2B (p<0.01). Serum glucose, triglyceride, low-density lipoprotein cholesterol and very low-density lipoprotein cholesterol levels were found to be increased and high-density lipoprotein cholesterol levels decreased in Group 2B (p<0.05). The intensities of iNOS staining in the retroperitoneal adipose tissue, COX-2 staining in the liver and both iNOS and COX-2 staining in the kidney were higher in Group 2B (p<0.05).

**Conclusion:**

Based on our findings, we believe that the offspring of dams which received a high fructose intake during their pregestation, gestation and lactation periods are at risk of developing metabolic syndrome in their later life only if they continue to receive a high intake of fructose. We therefore propose that the risk of developing metabolic syndrome can probably be reduced by modifying the diet of the offspring after weaning.

## INTRODUCTION

Countries tend to undergo nutritional transitions from traditional grain-based diets to high-fat high-sugar diets (1,2,3,4,5). The initial reports suggested that fructose altered the metabolism of glucose, lipid, uric acid and copper and that it also was a risk factor for cardiovascular disease (CVD) and hypertension (6,7,8). The hypothesis that excess fructose consumption is a cause of insulin resistance, obesity, elevated low-density lipoprotein (LDL) cholesterol and triglycerides, which are all factors leading to metabolic syndrome, has also been advanced in subsequent publications (3,9,10,11,12). Fructose came into consideration again through the high-fructose corn syrup (HFCS) hypothesis of Bray et al (1). These authors stated that the increase in consumption of HFCS has a temporal relation to the epidemic of obesity and that the overconsumption of HFCS in calorically sweetened beverages may play a role in the epidemic of obesity. This hypothesis has been a subject of extensive discussion, which all led to focus attention on HFCS as a unique cause of obesity (2,3,9,10). Recently, the Dietary Guidelines listed 5 significant diet-related chronic diseases faced by Americans: CVD (37% of the population), hypertension (34% of US adults), diabetes (nearly 11% of the population), cancer (41% of the population) and osteoporosis (50% of American women and 25% of men aged 50 years and over) (2). Fructose has been labelled as a risk factor for the first 4 of the above conditions and most recently also for nonalcoholic fatty liver disease.

Emerging evidence based on studies on animals and on observations has suggested that, through the mechanism of fetal/metabolic programming of adult-onset diseases, suboptimal nutrition experienced in early life enhances susceptibility to metabolic disorders which develop later in life (13). The results of many studies conducted on rat dams and in their offspring to explore the effects of maternal obesity and inappropriate maternal nutrition as risk factors for childhood and adult disease in the offspring have shown that excessive fructose intake during both gestation and lactation caused hyperinsulinemia and led tolterations in carbohydrate metabolism and in endocrine function (8,11,14,15,16). However, these studies have been conducted on rat fetuses and there is paucity of data on the long-term effect of fructose intake on the offspring of fructose-fed female rats when they become adult rats. We planned this study to evaluate the metabolic effects of ad libitum access to fructose solutions on the dams and on their offspring.

## METHODS

All procedures involving animals were conducted under license and in accordance with the Home Office Animals (Scientific Procedures) Act 1986 and were approved by the local ethics review committee.

Sixteen virgin female Sprague-Dawley rats (180-200 g; 8-10 weeks of age) were supplied by the Experimental Research Center of Gülhane Military Medical Faculty, Ankara, Turkey and were housed for 7 days for acclimatization. All animals were fed ad libitum standard laboratory chow, kept in a controlled temperature (20-22 ˚C) and humidity (55%-65%) environment and were subjected to a 12-hour light and 12-hour dark cycle. After acclimatization, the rats were divided into two groups: Group 1- regular diet (n=8), fed with standard laboratory chow and tap water; Group 2- fructose diet (n=8), fed with standard laboratory chow and tap water with 20% fructose (Sigma-Aldrich, St Louis, MO, USA). Consumed chow and water were also measured. Pre-weighed laboratory chow was provided. After 24 h, rats were briefly removed from their cages and weighed and the amount of food remaining, including any on the bottom of the cages, was recorded. All rats were fed with experimental diets ad libitum for at least 8 weeks before conception. All rats were mated two female-one male and they all were fed with their experimental diet during mating and pregnancy periods.

The dams were allowed to deliver spontaneously and they continued to be fed with their experimental diet ad libitum throughout the lactation period. Pups per dam were housed together to ensure adequate nutrition during lactation. At the end of the lactation period (approximately post-delivery 1 month), dams were fasted overnight; blood samples were collected from the dams under anesthesia and they were killed by cervical dislocation. Liver, kidney and retroperitoneal adipose tissue were harvested and fixed in 10% formaldehyde for histopathological examination.

After weaning, randomly chosen 2 female pups from each dam fed a regular diet (Group 1) were divided into two groups: Group 1A - regular diet (n=8), fed with standard laboratory chow and tap water and Group 1B - fructose diet (n=8), fed with standard laboratory chow and tap water with 20% fructose (Sigma-Aldrich, St Louis, MO, USA). The same experimental protocol was also applied to pups from dams on a fructose diet and they also were divided into two groups: Group 2A - regular diet and Group 2B - fructose diet. The rats were monitored with respect to their well-being, weight and their daily feeding schedule. The 20% concentrated fructose water was prepared daily. Puberty in these rats was assessed by vaginal opening at the postnatal 50th day, thus the study was terminated when the pups were fifty days old. At age 50 days, the rats were anesthetized; blood samples were collected by heart puncture and centrifuged for determination of biochemical parameters in the serum. Liver, kidney and retroperitoneal adipose tissue were harvested and fixed in 10% formaldehyde for histopathological examination.

Glucose, aspartate aminotransferase (AST), alanine aminotransferase (ALT), uric acid, triglyceride, total cholesterol, LDL, high-density lipoprotein (HDL) and very-LDL (VLDL) cholesterol levels were measured in the serum samples with an Olympus AU 2700 auto-analyzer (Olympus, Hamburg, Germany) using original kits.

### Pathology and Immunohistochemistry

Liver, kidney and retroperitoneal adipose tissue blocks from each subject were initially checked by hematoxylin-eosin (H&E)-stained sections to select the representative block for immunohistochemical staining. Four micron thick sections from each formalin-fixed paraffin blocks were immunostained using the primary antibodies against cyclooxygenase-2 (COX-2) (1:100, monoclonal, Neomarkers, Fremont, CA, USA) and inducible nitric oxide synthase (iNOS) (1:50, monoclonal, Neomarkers, Fremont, CA, USA). Immunohistochemistry was performed by the labeled streptavidin-biotin method, using the UltraVision Large Volume Detection System (LabVision, Fremont, CA, USA) kit. Presented immunohistochemical staining against iNOS and COX-2 indicate early phase of inflammation.

The immunohistochemical staining procedure was as follows: sections were deparaffinized and rehydrated in graded ethanol. After being rinsed in distilled water, sections were microwaved for 5 min at 600 Win 0.01 mol/L sodium citrate buffer (pH 6.0); this step was repeated three times. The slides were immersed in 3% H2O2 in distilled water for 5 min and then in blocking solution for 30 min to block endogenous peroxidase activity and unspecific binding sites, respectively. Sections were then rinsed in phosphate-buffered saline and incubated at room temperature with the primary antibody for 60 min, followed by rinsing in phosphate-buffered saline. Negative controls were performed by omitting the primary antibody. The sections were then treated with biotinylated secondary anti-rabbit antibodies in a dilution of 1:200 and antibody-binding sites were finally visualized by avidin-biotin peroxidase complex solution, using diaminobenzidine as a chromogen.

Immunohistochemically stained slides were reviewed by two blinded pathologists. The numbers of areas analyzed in tissues varied from 5 to 15 high-power fields per sample. The staining intensity was assessed semi-quantitatively. The immunohistochemical staining intensity was graded using a 4-stage grading scale: negative (0), weakly positive (1), moderately positive (2) and strongly positive (3).

### Statistical Analysis

All statistical analyses were carried out using SPSS statistical software (SPSS for Windows, Version 15.0, Chicago, IL, USA). Descriptive statistics were displayed as mean ± standard deviation or median (minimum-maximum). Comparison between two groups was performed using the t-test and comparison between 3 or more groups by the one-way ANOVA test. Bonferroni correction was used in post-hoc tests. The histopathology and immune histochemical analysis results were compared by Mann-Whitney U test. A p-value of <0.05 was considered as statistically significant.

## RESULTS

Consumption of chow and water, body weight in the two groups of female rats: While daily water consumption was significantly higher in Group 2 than Group 1 (53±16 mL/day vs. 28±12 mL/day; p<0.01), daily consumption of laboratory chow was significantly lower in Group 2 than Group 1 (11±3 g/day vs. 18±3 g/day; p<0.01) in the preconception period. Mean body weights of rats in the two groups were not statistically different in the period before conception (241±21 g vs. 238±24 g; p>0.05).

This pattern did not change during the lactation period. However, while the rats in Group 2 drank more fluid (67±26 mL/day vs. 35±21 mL/day; p<0.01), those in Group 1 ate more laboratory chow (18±3 g/day vs. 11±3 g/day; p<0.01). The mean body weight of rats in the groups were not statistically different at the end of the lactation period (248±25 g vs. 241±21 g; p>0.05).

Biochemistry in the two groups: These results are given in Table 1. There were no statistically differences between the two groups in terms of serum glucose, AST, ALT, uric acid, triglyceride, total cholesterol, LDL, HDL and VLDL cholesterol levels.

Consumption of chow and water and body weight in the offspring: After weaning, daily water consumption was significantly higher in Groups 2B and 1B (68±20 mL/day and 48±13 mL/day, respectively) when compared with Groups 1A and 2A (22±7.0 mL/day and 19±5.0 mL/day, respectively) (p<0.01). Daily laboratory chow consumption was significantly lower in Groups 2B and 2A (6.7±0.9 g/day and 6.2±0.8 g/day, respectively) than Group 1A and 1B (9.4±1.2 g/day and 10.2±1.4 g/day) (p<0.01).

The mean body weight of rats in Groups 1B and 2B (251±24 g and 216±29 g, respectively) was significantly higher than those in Groups 1A and 2A (169±18 g and 173±28 g, respectively) at the end of experiment period (p<0.01). Also, mean body weight was significantly higher in Group 2B as compared to Group 1B (251±24 g vs. 216±29 g; p<0.05).

Biochemistry in the offspring: These results are given in Table 2. While serum glucose, triglyceride, LDL and VLDL levels were higher, HDL levels were lower in Group 2B when compared with Group 1A and Group 2A (Group 2B vs. Group 1A and 2A; p<0.05). Serum triglyceride, LDL and VLDL levels were increased and HDL levels were decreased in Group 1B when compared with Group 1A.

Histopathological evaluation: The intensities of immunohistochemical staining for the harvested tissues of dams are summarized in Table 3. There are no statistically significant differences between dams in Groups 1 and 2 in terms of the intensity of COX-2 staining in the retroperitoneal adipose tissue, liver and kidney (p>0.05). However, iNOS intensity was increased in the retroperitoneal adipose tissue of dams fed with high-fructose diet (Group 1B vs. Group 1A; (p<0.05).

**Figure 1:**
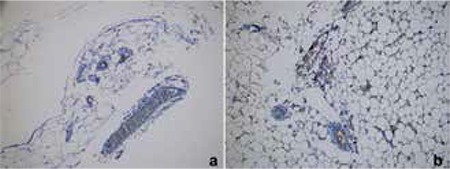
Inducible nitric oxide synthase staining of the retroperitoneal adipose tissue in Group 2B (a) vs. other groups (b)

**Figure 2:**
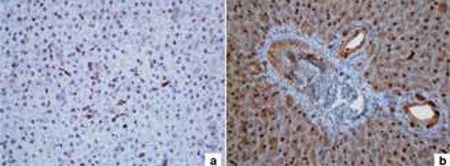
Cyclooxygenase-2 staining of liver tissue in Group 2B (a) vs. other groups (b)

**Figure 3:**
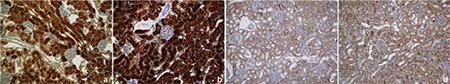
Inducible nitric oxide synthase staining of renal medulla and cyclooxygenase-2 staining of the glomerulus in Group 2B (a) vs. other groups (b)

The intensities of immunohistochemical staining for the harvested tissues of offspring are given in Table 4. The intensity of iNOS staining at the adipocyte, arteriole and venule of retroperitoneal adipose tissue was higher in Group 2B than in the other offspring groups (Group 2B vs. Group 1A, 1B and 2A; (p<0.01) (Figure 1). The intensity of COX-2 staining at the parenchyma, bile canalicula and venule of the liver was higher in Group 2B than the other offspring groups (Group 2B vs. Group 1A, 1B and 2A; (p<0.01) (Figure 2). The intensity of iNOS staining at the medulla and the intensity of COX-2 staining at the glomerulus of the kidney were higher in Group 2B than the other groups (Group 2B vs. Group 1A, 1B and 2A; (p<0.01) (Figure 3).

## DISCUSSION

In this study, we wanted to explore the effect of fructose intake on both dams and the female offspring of dams fed with a fructose diet. The first finding is that the 20% fructose diet intake before and during gestation does not cause hyperglycemia and hyperlipidemia in dams. The second finding is that offspring fed with a fructose diet, especially the offspring of dams on a fructose intake during gestation, gained more weight than the offspring of dams fed with a regular diet and that they were also hypertriglyceridemic. Finally, immunohistochemical evaluation revealed that while the retroperitoneal adipose tissue is intensely stained with iNOS, the liver is intensely stained with COX-2.

Our data showed that there was no evidence of metabolic syndrome in the dams consuming 20% fructose before, during and after gestational period; they displayed no changes in weight, serum glucose, triglyceride or uric acid levels. This observation was somewhat different from the reports indicating that increased fructose intake is associated directly with the development of diabetes and stating that metabolic syndrome is possibly due to increased production of uric acid and triglycerides (11,12,15,17,18). In animal studies, ats fed with 50% fructose diet during pregnancy and lactation demonstrated elevated plasma glucose concentrations and elevated liver gluconeogenic enzymes (19). A study conducted with a supraphysiological fructose diet (63%) showed that maternal fructose intake results in a fatty liver and glucose intolerance during pregnancy and lactation in the dams and to low birth weight in their offspring (20). Our results are somewhat in line with this study, although we did not find any difference in weight in groups fed a supraphysiological fructose diet. However, the studies applying a high-fructose intake do not reflect a generally consumed human diet. Based on this viewpoint, studies using a lower fructose level diet were performed and these also showed significant changes in related to fatty acid biosynthesis and carbohydrate metabolism (21). It was reported that a diet containing 10% fructose leads to maternal hypertriglyceridemia and altered maternal and fetal leptin signaling (17). Another study also reported that dams consuming an intake of a 10% fructose solution became hyperglycemic, hyperinsulinemic and hypertriglyceridemic (16). We fed the dams with 20% fructose and standard laboratory chow and there were no differences between them and dams fed with a regular diet. Possible explanation for this discrepancy could be due to differences in experimental design, or lower consumption of laboratory chow in rats exposed to fructose. Studies in rodents using very high fructose diets were commonly associated with significant reductions in overall food intake (22). This was also true in our study. Thus, we conclude that exposure to fructose during pregnancy might not be a contributing factor for metabolic syndrome by itself and factors such as overall food intake need also be taken into consideration. However, we believe that further investigations are needed to support this observation.

In this study, adult female offspring of dams fed with fructose showed evidence of mild metabolic syndrome; body weight was found to be significantly increased in adult female offspring fed with fructose diet regardless of the intake of their dams, whether they were on a regular or fructose diet. In addition, serum triglyceride and LDL-cholesterol level were increased, whereas HDL levels were decreased in these adult offspring. Hales and Barker (23) previously described and established a hypothesis which they called fetal programming, a hypothesis also termed as metabolic memory (24). This hypothesis states that a stimulus induced at a critical and delicate period of fetal development can lead to a long-term impact on the fetus. These authors also suggest that fetal malnutrition during pregnancy induces disruption in fetal growth and thinness at birth and programs the development of type 2 diabetes and metabolic syndrome in later life (25). Several studies have focused on this hypothesis and the effects of excessive carbohydrate consumption throughout the lactation period on the metabolic state of the offspring have been investigated by several groups. These experimental data on animal models showed that a high-fructose intake during pregnancy and lactation leads to metabolic dysfunction as well as to increased body weight, insulin resistance and retroperitoneal adipose tissue in both the dams and their newborns (22,24,26). However, our study differs from these studies since we further divided the female offspring of dams fed on a regular or fructose diet into two groups as those fed with a standard diet or a fructose diet. Based on our data, we propose that the risk of developing metabolic syndrome can be reduced by modifying the diet of the offspring after weaning.

There is limited experimental data showing whether the kidney of the offspring from dams fed with high-fructose diet is affected or not. Flynn et al (27) showed that high-fat/fructose feeding during prenatal and postnatal periods increases the severity of renal dysfunction in adult offspring. Jackson et al (28) also reported that combined exposure to overnutrition during fetal development and early postnatal development potentiate the susceptibility to renal disturbances later in life. We found that female offspring born from dams fed with a fructose diet during gestation and lactation and also fed with the same fructose diet for up to 8 weeks postnatally showed increased intensity of iNOS and COX-2 staining in their kidney, an effect which had never been investigated before. Adult offspring from dams fed a fructose diet during gestation and suckling followed by a regular diet post-weaning exhibited no changes in their kidneys. NO is produced by a reaction that is catalyzed by NOS. In the kidney, three isoforms of NOS, i.e. neuronal NOS, endothelial NOS and iNOS, have been identified. Intensified expression of iNOS has been detected virtually in all three cell types which resulted in the production of large amounts of NO with inflammation (29,30). COX-2, a key enzyme for prostaglandin biosynthesis, is absent in normal tissues but is rapidly induced during inflammatory reactions (31). COX metabolites have been implicated in functional and structural alterations in glomerular and tubulointerstitial inflammatory diseases (32). Our findings leads us to conclude that excessive fructose intake in rat dams causes inflammation in the kidney of their adult offspring.

Fructose intake during pregnancy and lactation increases the circulating fructose concentration in the dams and their offspring. Because ingested fructose can stimulate hepatic gluconeogenesis, it is possible that increased fructose in the fetal circulation, derived from maternal plasma fructose via the placenta, is associated with up-regulation of gluconeogenesis in the liver of the offspring. Experimental evidence indicates that the offspring of dams fed the junk-food diet during gestation and lactation developed higher rates of obesity, greater elevations in glucose and insulin levels and had an increased risk of fatty liver disease, as well as signs of steatosis and liver damage, when given free access to the same junk-food diet, compared with rats whose mothers had received a normal chow diet. In this study also, histopathological evaluation of the liver revealed increased intensity of COX-2 staining in the adult offspring of dams which had received an excessive intake of fructose during their gestation. Although we did not observe a fatty liver, we assume that exposure to an excessively high fructose intake during pregnancy, lactation and after weaning modulates the hepatic COX-2 levels, suggesting that fructose causes inflammation in the liver.

NOS and iNOS have been shown to be present in the white adipose tissue of the rat (33) and also in human subcutaneous adipose tissue (34), suggesting that this tissue may be a potential source of NO production. Moreover, it was shown that addition of NO resulted in decreased lipolysis in adipose tissue (35). We also showed that iNOS expression is increased at the retroperitoneal adipose tissue of the offspring of dams on a high intake of fructose. This result suggests an interaction between iNOS and fructose intake. However, this finding also needs further documentation by future studies.

In conclusion, we believe that fructose consumption before gestation, during pregnancy and lactation is not a contributing factor to induce metabolic syndrome by itself. Although it was proposed that fructose intake during pregnancy and lactation provides an environment for the fetus which leads to a change in fetal programming that influences the health risk of the offspring in his/her adult life, we suggest that the risk of developing metabolic syndrome can be reduced by modifying the diet of the offspring after weaning. We also think that the offspring of dams which received a high intake of fructose before gestation, during pregnancy and during lactation are at risk for developing the metabolic syndrome if they continue to be on a high-fructose intake.

## Figures and Tables

**Table 1:**
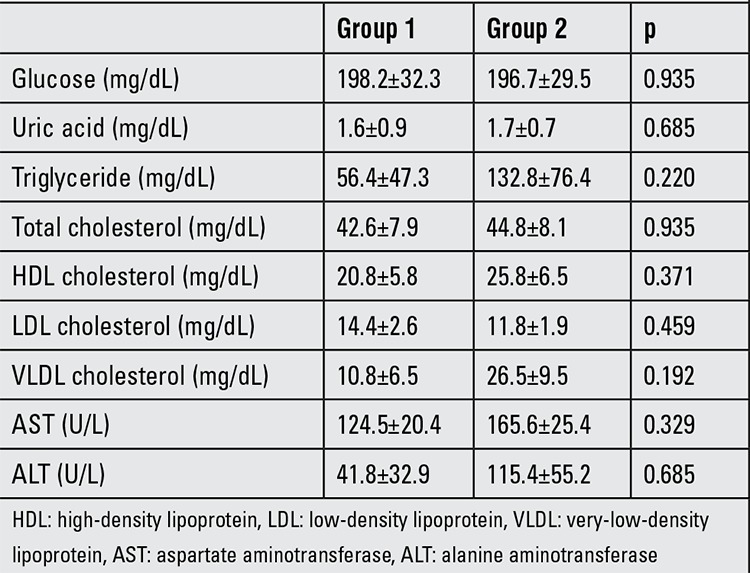
Biochemical values in dams

**Table 2:**
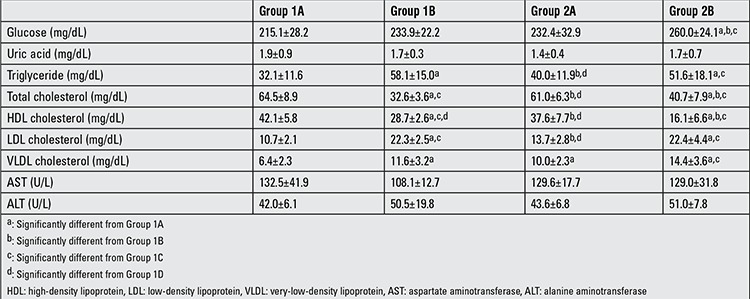
Biochemical values in the offspring

**Table 3:**
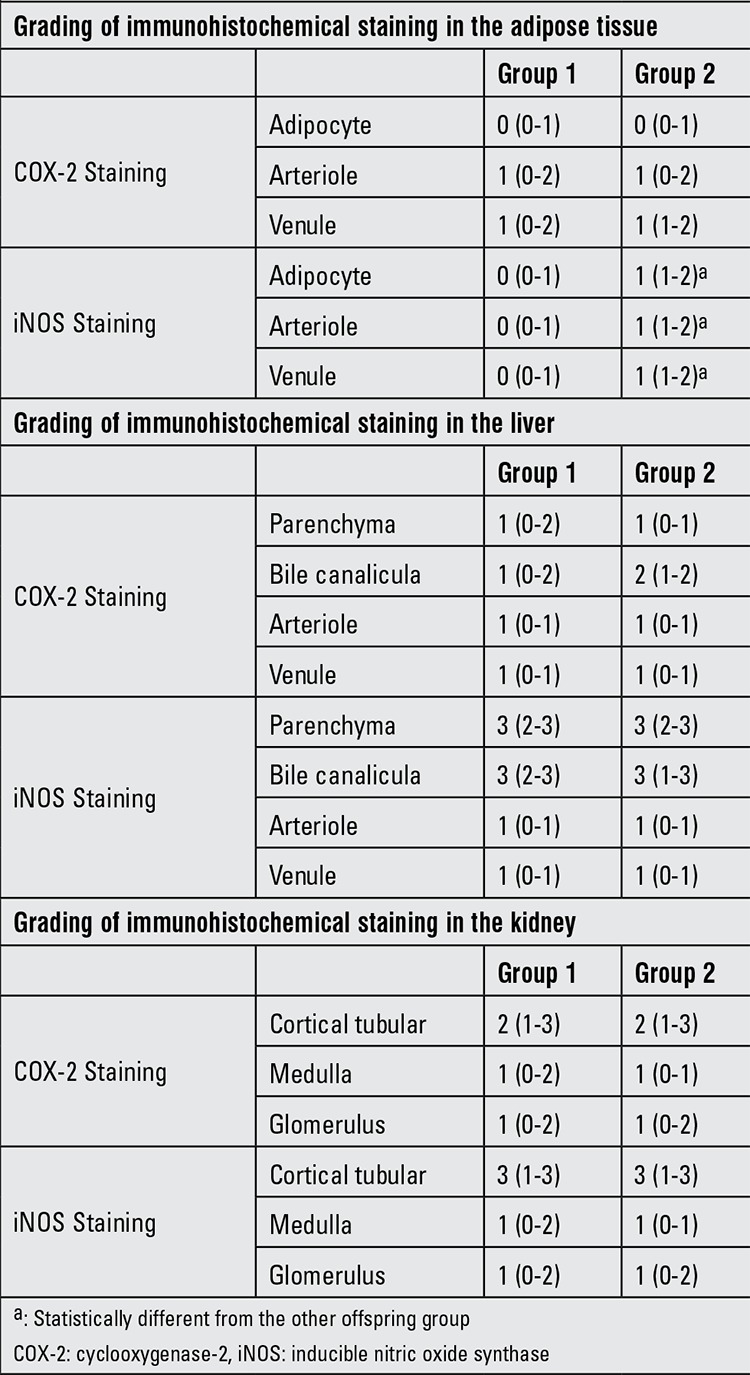
Immunohistochemical staining of three different tissue specimens in dams

**Table 4:**
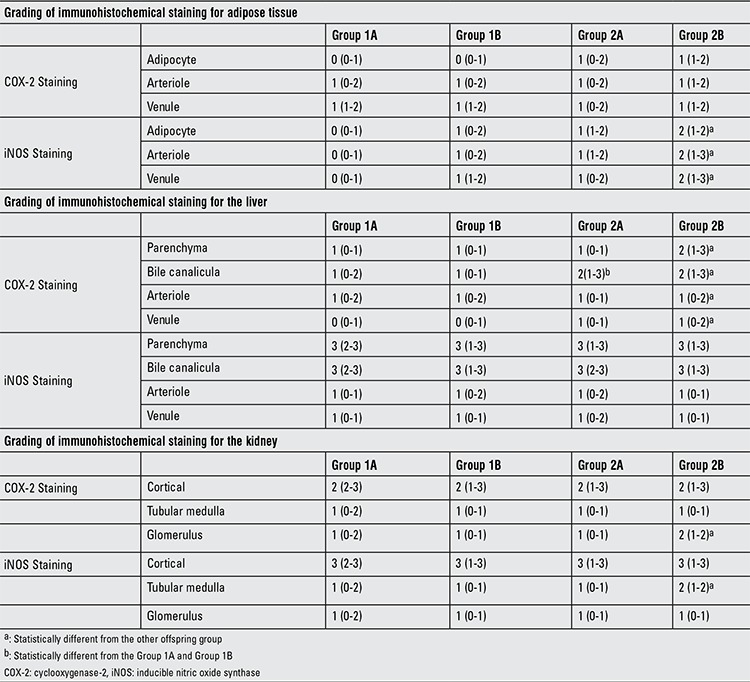
Immunohistochemical staining of three different tissue specimens in the offspring
